# Establishing the limits of efficiency of perovskite solar cells from first principles modeling

**DOI:** 10.1038/srep36108

**Published:** 2016-11-08

**Authors:** Oscar Grånäs, Dmitry Vinichenko, Efthimios Kaxiras

**Affiliations:** 1 John A. Paulson School of Engineering and Applied Sciences, Harvard University, Cambridge, Massachusetts 02138, United States; 2 Department of Physics and Astronomy, Division of Materials Theory, Uppsala University, Box 516, SE-75120 Uppsala, Sweden; 3 Department of Chemistry and Chemical Biology, Harvard University, Cambridge, Massachusetts 02138, United States; 4 Department of Physics, Harvard University, Cambridge, Massachusetts 02138, United States

## Abstract

The recent surge in research on metal-halide-perovskite solar cells has led to a seven-fold increase of efficiency, from ~3% in early devices to over 22% in research prototypes. Oft-cited reasons for this increase are: (i) a carrier diffusion length reaching hundreds of microns; (ii) a low exciton binding energy; and (iii) a high optical absorption coefficient. These hybrid organic-inorganic materials span a large chemical space with the perovskite structure. Here, using first-principles calculations and thermodynamic modelling, we establish that, given the range of band-gaps of the metal-halide-perovskites, the theoretical maximum efficiency limit is in the range of ~25–27%. Our conclusions are based on the effect of level alignment between the perovskite absorber layer and carrier-transporting materials on the performance of the solar cell as a whole. Our results provide a useful framework for experimental searches toward more efficient devices.

Photovoltaic applications of metal halide perovskite absorbers face a number of outstanding challenges, including materials stability, hysteresis effects of the current-voltage characteristics, and fine-tuning of the absorption properties^1^,^2^. The perovskite ABX_3_ structure, where A and B are typically organic or inorganic cations, and X is an oxygen or halogen anion, offers a large chemical phase space, allowing many properties to be tailored, albeit not independently. We argue that reaching the ideal efficiency, apart from optimizing the quality of materials and interfaces, is more challenging than optimizing the photo absorber; when absorption properties are tailored by adjusting the composition, band alignment and effective masses are also affected.

To establish the theoretical limits of efficiency, we assume ideal interfaces and defect-free crystals. We investigate how the presence of different ions on the A, B and X site of the perovskite structure impacts the photovoltaic performance, using electronic structure calculations and available experimental information, together with thermodynamic considerations. We show that the level alignment to the electron- and hole-transporting media (ETM and HTM) is central to reaching maximum efficiency for heterogeneous positive-intrinsic-negative (PIN) junctions. In fact, the efficiency limit for many of the perovskites is similar, in the ~25–27% range, given perfect band-alignment to ETM and HTM. Current state-of-the-art cells reach >80% of the theoretical maximum efficiency, indicating that higher performance is mostly a matter of interface engineering and the construction of multi-junction cells. Our results shed light into the performance differences arising from different perovskite compositions, choices of electron and hole transporting media or modification of the heterojunctions^3^,^4^. Thus, they serve as a guide to further work on what HTM and ETM are suitable for optimal device performance.

## Model of the solar cell

**Figure 1:**
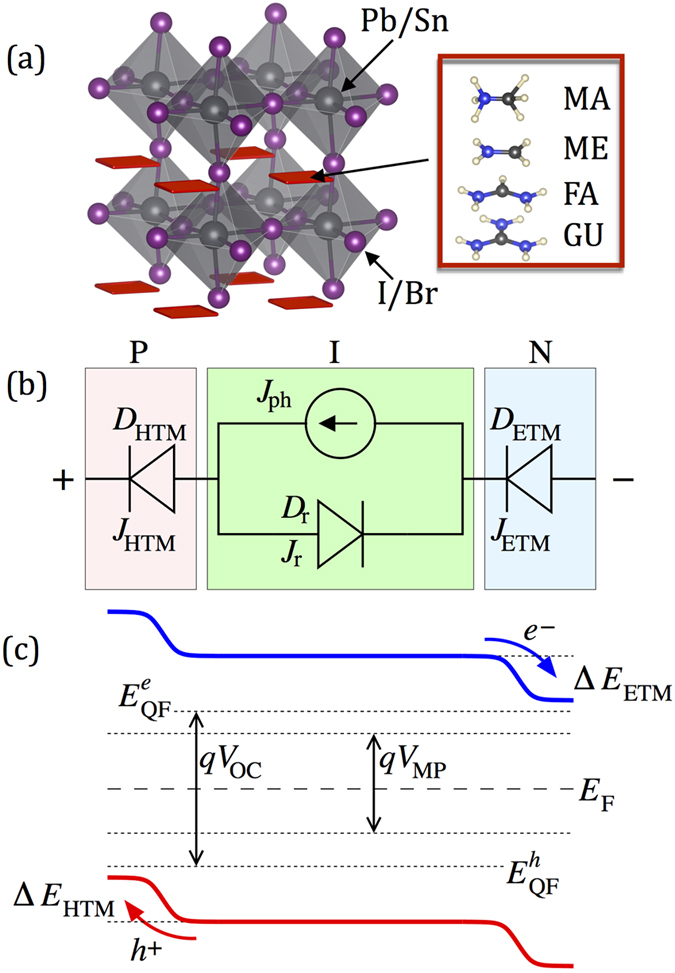
(**a**) Composition of perovskites under consideration (in the organic cation, blue = N, black = C, white = H). (**b**) Equivalent circuit of the PIN photo absorbing heterostructure. J_ph_ denotes the photon induced current, D_r_ the intrinsic diode (additional diode currents from Shockley-Reed-Hall recombination can be added), D_ETM/HTM_ represent rectifying diodes, the diode voltage drop, as well as forward direction is determined by the energy level alignment. (**c**) Schematic overview of the energy level diagram of the intrinsic part, with band offsets ΔE_HTM_, ΔE_HTM_ to ETM/HTM. *E*_*F*_ is the position of the Fermi level, 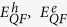 the quasi Fermi-levels under illumination with open circuit, *V*_*MP*_ the voltage for the maximum power point and *V*_*oC*_ the open-circuit voltage.

We aim to establish theoretical limits for the power conversion efficiency (PCE) of the perovskite-based solar cells as a function of chemical composition of the perovskite layer and the electronic properties of the electron- and hole-transporting media, by using the thermodynamic approach pioneered by Shockley and Queisser^5^. Recent experiments indicate that exotic effects such as ferroelectricity are not responsible for the reported high efficiencies^6^,^7^. The most important properties of the perovskites as photo-absorbers are the resilience to form recombination centres and the reasonable interface quality to many ETMs and HTMs. Thus, it is reasonable to determine efficiency limits of perovskite solar cells from arguments based on detailed balance. Experimental characterization of the heterojunctions suggests that perovskite solar cells are of PIN character^8^,^9^, where the perovskite itself comprises the central intrinsic semiconductor. We model the cell as a PIN heterojunction, where the carriers are generated by photon absorption in the intrinsic perovskite layer, separated and injected across a corresponding interface into the electron- or hole-transporting medium. We model interfaces between the perovskite and carrier transporting media as diodes *D*_ETM/HTM_ with the constant voltage drop (CVD) approximation, that is, we ignore the band matching character. We also ignore back transfer of carriers, which has been experimentally proven a slow process^10^. We present in Fig. 1(b) a schematic of the circuit. We assume a constant-entropy mode of operation; with that, the electrochemical potential of the cell can be expressed as:


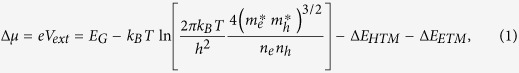


where *E*_*G*_ is the perovskite absorber band gap, 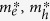 are carrier effective masses, *n*_*e*_, *n*_*n*_ are carrier densities, and Δ*E*_*HTM*_, Δ*E*_*HTM*_ are the potential drops at the boundaries between the perovskite layer and carrier-transporting media. The energy levels in our model are shown in Fig. 1(c). We ignore shunt and series resistances, and consider only radiative recombination to provide the upper limit for experimental efforts. With these assumptions, the current is given by *J* = *e*(*N*_*ph*_ − *RR*(*V*_*ext*_)), where *N*_*ph*_ is the density of absorbed photons, and *RR*(*V*_*ext*_) is the rate of radiative recombination:


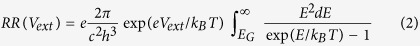


In order to compute the PCE, we use the NREL reference AM1.5 spectrum normalized to 1 kW/m^2^. The maximal extractable power density is determined by maximizing 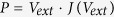. From these considerations, we can identify three main components that determine the efficiency of the perovskite-based solar cell: the band gap of the absorber, the carrier characteristics relevant for entropic contribution, and the alignment between absorber and carrier transporting media. In the following we discuss our approach to determining these quantities using a combination of first-principles modelling and available experimental data.

The band gap of the perovskites is generally direct, or close to direct^11^. Together with band edge characters that allow for dipolar transitions this leads to an extraordinarily high optical absorption coefficient, which allows for a thin-film cell architecture. The onset of absorption is determined by the band gap, which is crucial for estimating the fraction of absorbed photon flux.

In the context of first-principles electronic structure calculations with density functional theory (DFT), due to incorrect Coulomb interaction asymptotes in semi-local functionals^12^, the band gap is underestimated; for the hybrid organic-inorganic perovskites the error between different DFT calculations is fairly constant (for instance, the difference between the gaps of MASnI_3_ and MAPbI_3_ is only 0.07 eV, as inferred by comparison with GW results)^13^,^14^,^15^. In order to obtain realistic band gaps for the compounds studied here, we use MAPbI_3_ (for which reliable experimental data is available) as the reference material and calculate the difference in the DFT computed gap with all other compounds. We employ two different methods for calculating the shifts in band gap of the perovskites under consideration, the results ranges between 1.2 eV and 2.1 eV (in PBE) or between 0.9 eV and 2.3 eV (in HSE).

Experimentally, the photo-induced excitons dissociate rapidly into free carriers due to low exciton binding energy of 10–50 meV^16^,^17^; for this reason it is not necessary to model excitonic effects.

**Figure 2:**
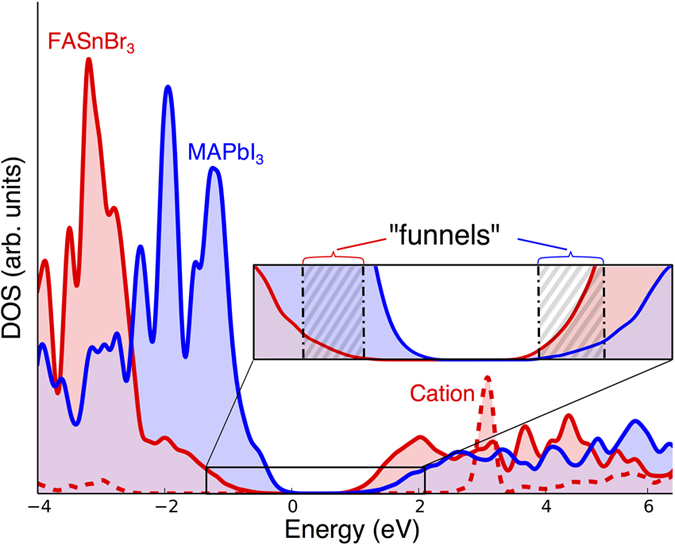
Densities of states for methylammonium lead iodide (MAPbI_3_) and formamidinium tin bromide (FASnBr_3_), showing contributions from the organic cation and the inorganic sublattice. The band edges are spanned by metal and halogen states, with the cation having only minor hybridization. The inset shows the shallow DOS (‘funnel’) near the VBM, characteristic for tin-based compounds, and shallow DOS near the CBM, typical of Pb-based compounds (see text for details).

Charge mobility is a very important factor for efficient solar cells and is key to the high reported efficiencies. Long mean free paths have been observed in experiments: early work measured over 1 *μ*m for polycrystalline samples^9^ while more recent work reported values up to 175 *μ*m under illumination and 3 mm under weak light^18^. Apart from the diffusion length, the low carrier masses decrease the entropic loss of the open-circuit voltage. Small masses are manifested as funnel-like structures on the electronic density of states, with a minimal number of states at the band edges^19^. The perovskite compounds have a pronounced funnel effect (see Fig. 2), which contributes to the high cell efficiency at operating temperatures. We compute the average of the effective mass tensor of the carriers from the band structure using a high density (approximately 2 × 10^7^ k-points/Å^−3^) mesh. Finally, we determine the entropy contribution of the excited carriers to the quasi Fermi level splitting using an effective density of excited states of 10^15^ cm^−3^. The carrier masses of the compounds under consideration are smaller than, for example, Si which results in a smaller effect of the temperature arising from carrier entropy and a smaller deviation from the Shockley-Queisser limit^20^. The results show a clear trend: Pb compounds have lower conduction band mass and higher valence band mass in relation to the Sn counterparts. The effective masses range between 0.1 and 0.3 in units of m_e_.

Properties like the natural level alignment and the absorption onset are difficult to measure from an experimental point of view^21^. Interface and surface dipoles, defects and sample inhomogeneities result in substantial differences in available experimental data. For example, the depletion layer is reported to be somewhere between 45 to 300 nm^22^,^23^, indicating that level alignments play a crucial role for the carrier concentrations and for the potential gradient in the perovskite. The calculation of the natural band alignment from first principles using core levels or by inspecting how the average electrostatic potential changes on a site in different environments has been treated extensively in the literature^24^,^25^. We employ a method of alignment similar to what was first suggested by Massidda *et al*.^26^. and later refined by Wei and Zunger^27^, and by Lang *et al*.^28^. We estimate the relative positions of valence and conduction bands of perovskites with different composition using the average electrostatic potential on the B site ion, or, in the case of B-ion substitution, on the X-site ion. We also use the equivalent formalism of core-levels as a test for consistency. The band alignments, from the relative position of band edges to those of MAPbI_3_, are used to map the corresponding potential drops for the rectifying diodes appearing at the heterojunctions between the perovskite and the charge-transporting media. We do not explicitly model the P and N materials; instead, we map out the efficiency as a function of the natural band alignment with respect to MAPbI_3_. Recent computational work provided estimates for the potential alignment of a number of common semiconductors in relation to MAPbI_3_; with our results, this can be used directly to estimate how the efficiency is altered^29^.

MAPbI_3_ has a cubic structure at high temperature and undergoes a transition to a tetragonal structure close to room temperature. The two phases are known to co-exist to much lower temperatures. To reduce the complexity of calculating the level alignments, we concentrate on the cubic phase of the perovskite cell. This involves calculating: the core-level (CL) alignment in the super cell between the left (L) and right (R) perovskites; the CL alignment to valence band maxima (VBM) in the *strained* materials (with strain according to the relaxed heterostructure); the CL alignment to VBM in the relaxed structure. To account for possible steric effects from the orientation of the organic cations, we consider them oriented in the plane orthogonal to the super cell stacking, which resembles the tetragonal phase. This procedure allows us to determine the natural band alignment between MAPbI_3_ and the other perovskites under consideration. Results for all compounds are available in the Supplementary Material. Reviewing other theoretical approaches reveals a significant spread in reported data^30^,^31^, but the trends are consistent with our findings. Previous work on GaAs/AlAs heterojunctions estimated errors in absolute value on the order of 0.05 eV^26^. For the calculations reported here, we expect slightly larger errors due to the complex symmetry breaking by the organic cation. Previous studies using similar methods but without taking into account strain effects reported similar values of band alignment for the compounds under consideration^32^.

In cases where an increase (decrease) in the potential for electrons (holes) occurs, the voltage drop over the junction depends exponentially on the barrier height, with most of the external bias over the junction. In these cases, the quasi Fermi-level splitting goes to zero, inducing strong recombination in the junction, and the value of J_sc_ effectively goes to zero as well. An accurate treatment of the junctions requires knowledge of the dielectric properties and density of states of specific HTMs and ETMs.

## Results and Discussion

We have investigated 16 hybrid organic-inorganic compounds with the perovskite structure of the general composition ABX_3_. For A, we have considered 4 organic cations, the traditionally used methylammonium, and three other ions of similar size and conjugated *π* system of increasing size: methyleneiminium (ME = 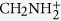), formamidinium (FA = 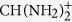) and guanidinium (GU = 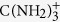). We consider Pb^2+^ and Sn^2+^ ions for the B position, and Cl^−^ and Br^−^ ions for the X position. The most important feature of the electronic structure of these compounds is that, regardless of the size of the *π* system of the A cation, the band edges are spanned by *s-* and *p-*states of the B ion and by *p-*states of the X ion. The A ions act mostly as spacers and affect the electronic structure and properties of the perovskite through the changes they induce in lattice structure, as we discuss below.

The bottom of the conduction band is spanned mostly by the *p*-states of B ions with an admixture of *p*-states of X-ions. There are two factors in action when the B site is changed: the relative electronegativity of Pb and Sn, and the strength of spin-orbit coupling, which leads to splitting of the bottom of the conduction band. Those factors lead to lowering of the absolute position of the CBM for Sn compared to Pb. Moreover, due to stronger spin-orbit coupling in Pb, the CBM splitting is larger, which leads to higher curvature of the CBM and, therefore, to lower effective masses for electrons in Pb-based perovskites (~0.1 m_e_) compared to Sn-based perovskites (~0.2–0.3 m_e_). With Br in the X position the CBM is higher due to the more covalent character of the bonds and larger splitting between bonding and antibonding orbitals.

The top of the valence band consists of an antibonding combination of X *p*-states and B *s*-states leading to a lower absolute position of the VBM with increasing electronegativity of the X ion. Substitution of Pb by Sn on the B site leads to an increase of the VBM energy due to larger overlap of the Sn *s*-states with the X *p*-orbitals. An increase in the size of the A cation leads to a decrease in the VBM energy due to expansion of the lattice structure from the decrease in overlap between the *s*-orbitals of B and the *p*-orbitals of X, and the concomitant lowering of the antibonding level energy. Smaller overlap between the *s*-orbitals of B and the *p*-orbitals of X also leads to a less dispersive band and an increase in the effective carrier mass for the holes for Pb (~0.15–0.3 m_e_) compared to Sn (~0.07–0.1 m_e_). A similar effect is introduced by increasing the size of the A cation. Another factor is the Darwin term effect on the *s*-orbitals of B ion levels, which is larger for Pb, resulting in flatter bands.

**Figure 3:**
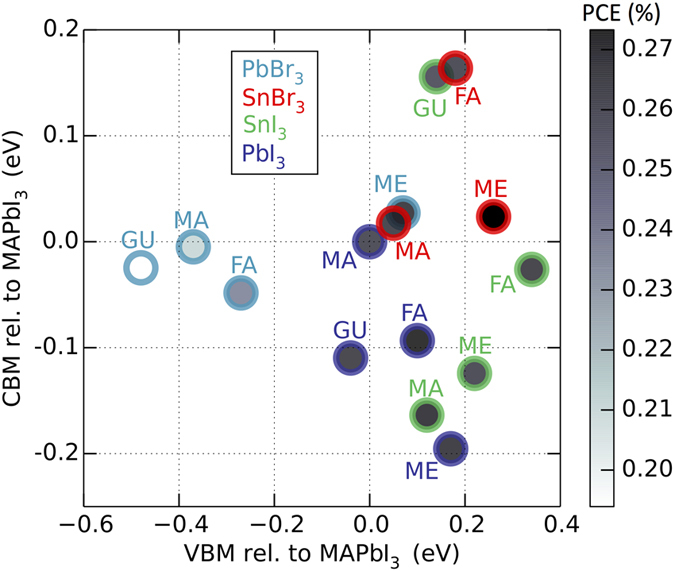
Level alignment in relation to MAPbI_3_ in eV. Classes of compounds are colored according to their inorganic backbone: PbI_3_ = purple, PbBr_3_ = blue, SnI_3_ = green, and SnBr_3_ = red. The PCE determine the darkness of the fill color in the circles. Note that the spread in VBM is much larger than the spread in CBM. Detailed numerical results are available in the Supplementary Material, Tables 4 and 5.

From the absolute positions of the CBM and VBM we obtain the value of the band gap, which is in the 1.1–1.6 eV range for most compounds except for FA-, MA-, and GU-based perovskites with PbBr_3_ lattice backbone. Based on our calculations, we conclude that all of the compounds studied (except for those mentioned above) demonstrate power conversion efficiency of 25–27% regardless of actual chemical composition, provided optimal band alignment is satisfied. This clearly demonstrates that the intrinsic properties of hybrid organic-inorganic compounds, that is, band gap and carrier effective masses, are not the limiting factor in determining the efficiency. Rather, it is the misalignment of the absolute positions of the band edges that can lead to substantial performance deterioration (about 5% PCE for every 0.2 eV of mismatch, see Supplementary Material Fig. 3). We map out the band edge positions, which are equivalent to the optimal carrier-transporting material levels, and the PCE achieved with optimal level matching in Fig. 3. Our overall conclusion is that experimental efforts should be directed towards optimization of the device as a whole, using as a guide the results outlined above.

In particular, the chemical composition of the perovskite has a profound effect on the position of VBM and CBM levels: an increase in the size of the A ion leads to lowering of the VBM; compounds with Pb on the B site have lower VBM and higher CBM compared to Sn-based perovskites; Br-based compounds have lower VBM and higher CBM compared to I-based ones. These principles can be used to tailor the composition of the perovskite absorber in order to optimize the efficiency of the cell overall by ensuring optimal level matching with the carrier-transporting materials.

## Methods

### First principles calculations

The DFT simulations are performed using the PBE^33^ exchange-correlation functional with van der Waals corrections^34^,^35^, as implemented in VASP^36^,^37^,^38^,^39^. We also employ the HSE hybrid density functional^40^ for an independent estimate of the relative shifts in band edges. We use a Г-centered k-point grid of 8 × 8 × 8 and a plane-wave energy cutoff of 550 eV. The SCF loop convergence is halted when the energy difference to previous steps is 10^−5^ eV, structural relaxations are stopped when the energy difference to the previous geometry is 10^−3^ eV. For structural properties integration over the occupied states is performed using Gaussian smearing with a width of 0.1 eV. For core-levels and average potentials the modified tetrahedron method was employed. Further details can be found in the Supplementary Material.

### Circuit modelling

The model of the cell is implemented in Python and solved using the NumPy package^41^. The graphics is made using matplotlib^42^.

## Additional Information

**How to cite this article**: Grånäs, O. *et al*. Establishing the limits of efficiency of perovskite solar cells from first principles modeling. *Sci. Rep.* **6**, 36108; doi: 10.1038/srep36108 (2016).

**Publisher’s note:** Springer Nature remains neutral with regard to jurisdictional claims in published maps and institutional affiliations.

## Supplementary Material

Supplementary Information
