# Supplementary material for: Establishing the limits of efficiency of perovskite solar cells from first principles modeling
Source: Sci Rep. 2016 Nov 8;6:36108. doi: 10.1038/srep36108 (PMC5099916; doi:10.1038/srep36108)
Supplement: Supplementary Information [file srep36108-s1.pdf]

# Establishing the limits of efficiency of perovskite solar cells from first principles modeling

Oscar Grånäs[1,2], Dmitry Vinichenko[3] and Efthimios Kaxiras[1,4]

1) John A. Paulson School of Engineering and Applied Sciences, Harvard University, Cambridge, Massachusetts 02138, United States
2) Department of Physics and Astronomy, Division of Materials Theory, Uppsala University, Box 516, SE-75120 Uppsala, Sweden
3) Department of Chemistry and Chemical Biology, Harvard University, Cambridge, Massachusetts 02138, United States
4) Department of Physics, Harvard University, Cambridge, Massachusetts 02138, United States

# Supplementary Material

## Details of electronic structure calculations

### *Structural properties*

All *ab-initio* calculations are performed using the Vienna Ab-Initio Simulation Package (VASP).[1-4] The structures are optimized using density functional theory, employing the exchange correlation functional by Perdew *et al.* (PBE).[5] Van der Waals interactions were previously shown to be important,[6] so we included dispersion interactions using the scheme developed by Tkatchenko *et al.* [7,8]. Spin-orbit coupling, although not crucial for the structure, is included in all calculations. The Brillouin zone is sampled with a Γ-centered mesh of 8x8x8 k-points. All calculations are performed with a plane-wave energy cut-off of 550eV. The lattice parameters are reported in Table 1.

| | Lattice Parameter (Å) | | | |
|---|---|---|---|---|
| | $PbI_3$ | $SnI_3$ | $PbBr_3$ | $SnBr_3$ |
| ME | 6.28 | 6.17 | 5.91 | 5.81 |
| FA | 6.37 | 6.30 | 6.01 | 5.94 |
| MA | 6.32 | 6.23 | 5.93 | 5.85 |
| GU | 6.45 | 6.40 | 6.11 | 6.18 |

**Table 1. Lattice parameter of the cubic structure for all compounds.**

| Bond distortion (%) | | | | |
|---|---|---|---|---|
| | $PbI_3$ | $SnI_3$ | $PbBr_3$ | $SnBr_3$ |
| ME | 0.3 | 0.8 | 0.7 | 0.6 |
| FA | 1.2 | 2.2 | 1.8 | 2.2 |
| MA | 1.8 | 0.7 | 1.7 | 1.0 |
| GU | 0.7 | 4.6 | 1.4 | 13.2 |

**Table 2. Mean relative displacement of the B-X bond. Low numbers indicate stable octahedron; high numbers indicate instabilities. Due to the non-spherical organic cation the bonds are slightly distorted in all cases.**

A hand waving measure of stability against distortions of the octahedral cage can be obtained by allowing full relaxation of all parameters of the unit cell. Table 2 displays the relative bond distortion in the octahedron. The standard deviation of the B-X-bond lengths should be low if the octahedron is stable. Our calculations show that $GUSnBr_3$ is most likely unstable as a result of the unfavorable size ratio of the GU molecule to the rather small Sn and Br ions. $GUSnBr_3$ is omitted from subsequent calculations due to the low likelihood of producing the compound in the pseudo-cubic structure.

Visualizations and manipulations of the crystal structure is done with the software packages VESTA and cif2cell [9,10].

*Band structure and effective carrier masses*

Valence and conduction band masses are calculated using a fixed charge density, with a dense k-point mesh around the R-point in the Brillouin zone. The band masses are converged at a sampling density of approximately $2\times10^7$ k-points per $Å^{-3}$. The masses reported are well in line with recent experimental work [11] slightly lower than what is

reported in other calculations [12-14]. In modelling the efficiency, only its temperature dependence is affected by the effective masses.

The density of states for all compounds is shown in Fig. 1, these calculations where performed with a Γ-centered mesh of 12x12x12 k-points using the hybrid DFT functional by Heyd, Scuseria and Enzerhof (HSE06). [15]

| | Valence band masses | | | | Conduction band masses | | | |
|---|---|---|---|---|---|---|---|---|
| | $PbI_3$ | $SnI_3$ | $PbBr_3$ | $SnBr_3$ | $PbI_3$ | $SnI_3$ | $PbBr_3$ | $SnBr_3$ |
| ME | 0.10 | 0.07 | 0.13 | 0.06 | 0.10 | 0.31 | 0.13 | 0.31 |
| FA | 0.20 | 0.07 | 0.21 | 0.18 | 0.09 | 0.17 | 0.11 | 0.16 |
| MA | 0.27 | 0.08 | 0.34 | 0.17 | 0.08 | 0.15 | 0.11 | 0.14 |
| GU | 0.33 | 0.15 | 0.33 | | 0.10 | 0.20 | 0.12 | |

**Table 3. Effective masses for conduction band (CB) and valence band (VB) calculated using a polynomial fit to a high density k-point grid around the R-point in the Brillouin zone.**

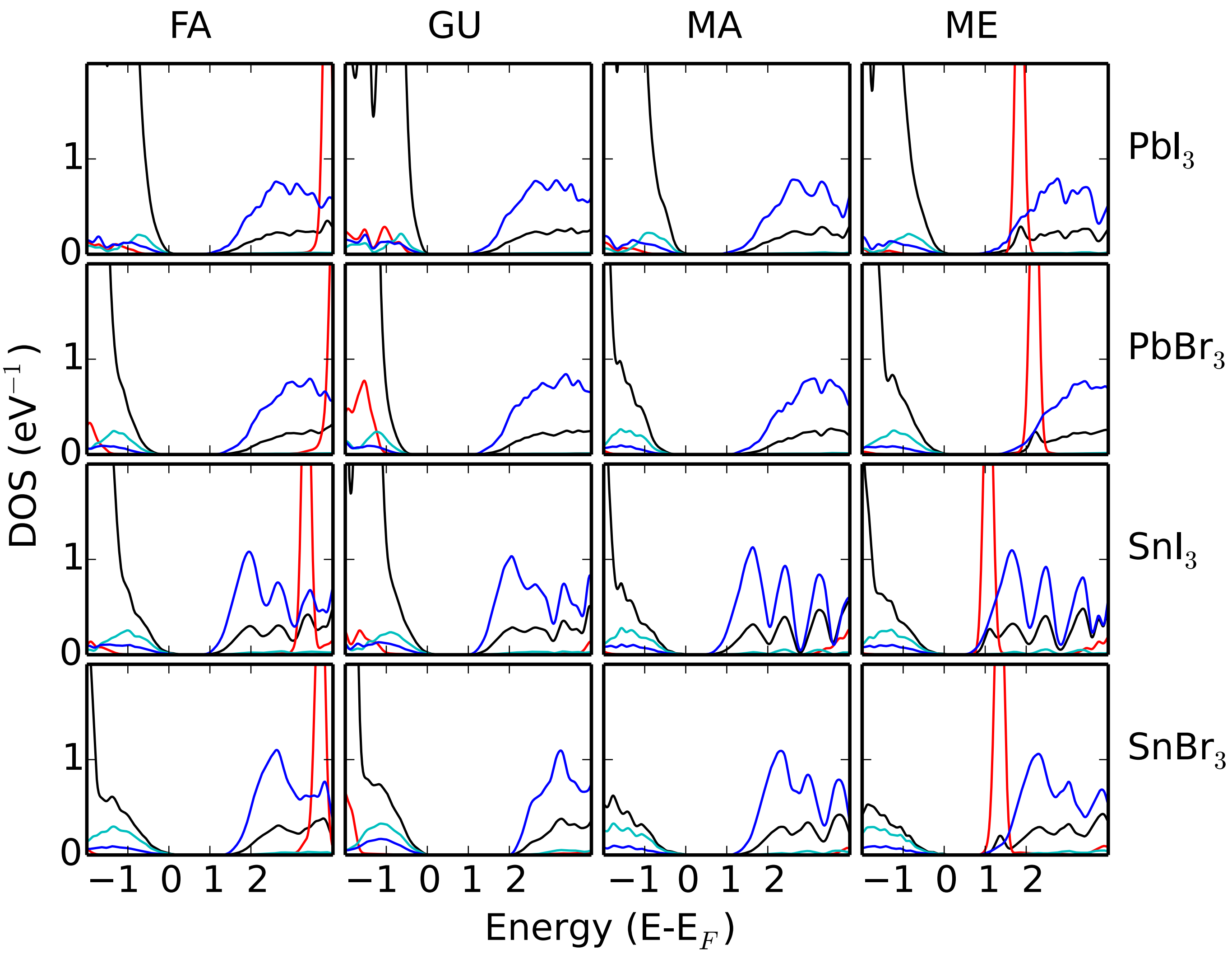


**Figure 1. Density of states for all compounds, calculated with the HSE06 functional. Columns are ordered by the organic (A) ion, and rows by the inorganic sub-lattice. Red curves correspond to states on the A-site ion, black to X-site, cyan and blue corresponds to s- and p-states on the B-site ion.**

*Supercells for level matching*

In order to determine the relative shifts in valence and conduction band between the perovskites we use a supercell approach. Five calculations for each pair of compounds has to be considered. First, the super lattice, setup according to figure 2, center $(ABX_3)_L/(ABX_3)_R$. In this structure we calculate the difference in average potential (AP) on the common ion (B or X, depending on substitution), we also double check using the core-level (CL) in a similar fashion. The core-level results show some dependence on what core-state is chosen, as a representative example the relative natural band

alignment from $MAPbI_3$ to $MAPbBr_3$ is -0.37 eV from the average potentials and -0.33 eV, -0.37, -0.41, -0.53 eV from the 1s, 3p, 4d and 5d core-states on Pb respectively. Due to the lattice mismatch between the two materials there is no obvious better choice for lattice parameter, hence the shape of the super lattice is relaxed. The relative positions of the AP/CL for the two materials within the supercell can now be calculated. The strain on the constituents induce a change in the AP/CL to valence band maxima that has to be accounted for. As the compound is pseudo cubic, it is straightforward to setup a unit cell of the strained material from the center of the left and right sub-units of the superlattice, and subsequently calculate the change in AP/CL to valence band maxima with respect to the relaxed pure compounds. Care has to be taken with respect to change of the B-ion, hence, where we have a common X-ion. Since many of the organic cations are polar, this induces possible complications when determining the average potential on the X-ion, due to the 3 different crystallographic sites. We use the X-ion situated on the face between the planes in which the organic cations dipole is oriented. Finally, when the strained cell AP to VBM is known, we can calculate the corresponding parameters for the relaxed cell. We now have access to the full relative difference, the natural band alignment, of the two constituents of the superlattice using the formula:

$$\Delta E_{VBM}^{LR} = E_{VBM}^{R} - E_{VBM}^{L} = ((E_{VBM-Core}^{R})_{GS} - (E_{VBM-Core}^{R})_{Strain}) - ((E_{VBM-Core}^{L})_{GS} - (E_{VBM-Core}^{L})_{Strain}) + (E_{Core}^{L} - E_{Core}^{R})_{Superlattice}$$

The shift in CBM can now be calculated by inspecting the change in valence band maxima and band gap. Note that the actual value of the band gap will be substantially underestimated for both PBE96 and HSE06, but we are only interested in the relative

shift. The values are related to $MAPbI_3$, for which we assume the experimental band-gap of 1.6eV. We employ both the PBE density functional and the HSE06 hybrid functional to check consistency of the trends. More expensive methods such as GW can give more reliable shifts when applied with great care, but still omits effects of zero-point motion to the gap. Previous work using different levels of $G_xW_x$ shows substantial differences in the reported band gaps, indicating the difficulties involved in these calculations.[14,16]

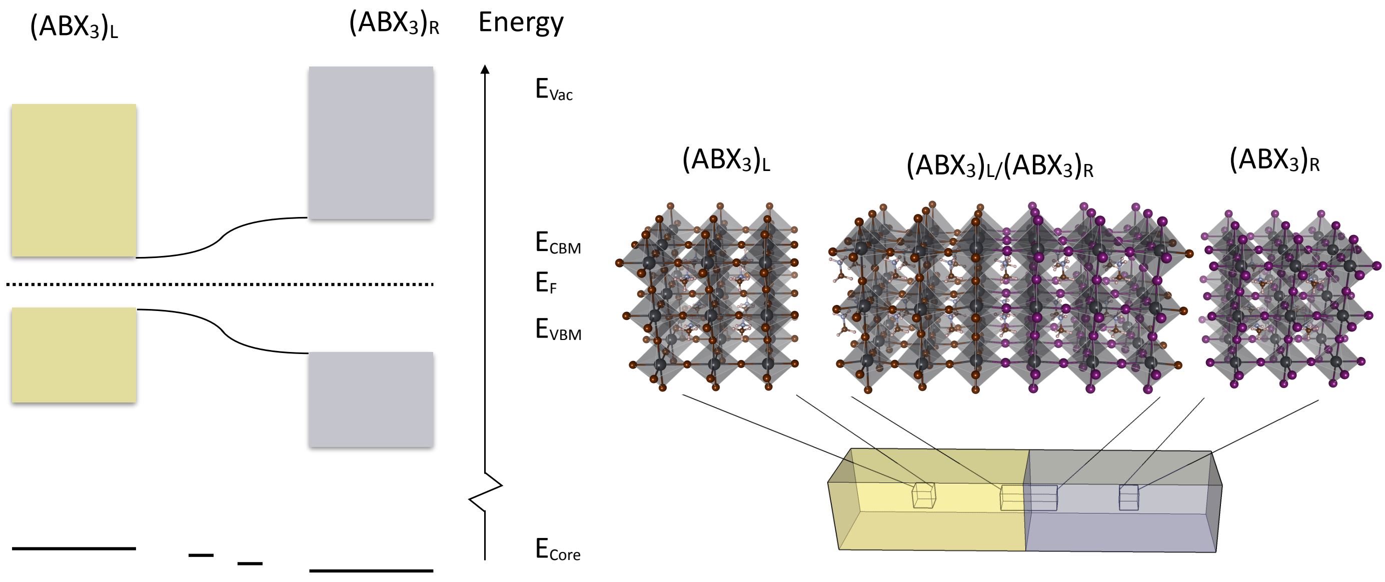


**Figure 2. Schematic diagram of energy levels across a heterojunction (left). Schematic picture of the interface in a heterojunction (right). The three geometries calculated are the interface, strained single material and ground state of the single material. At the interface we calculate the relative position of the core-states (and the relative difference of the average electrostatic potentials), in the strained material we calculate the position of the valence band maxima in relation to the core-levels, finally we estimate the effect of the strain by calculating the position of the valence band maxima relative to the core-states in the ground state.**

| | $PbI_3$ | $SnI_3$ | $PbBr_3$ | $SnBr_3$ |
|---|---|---|---|---|
| **Valence band alignment (PBE/HSE)** | | | | |
| ME | 0.17 | 0.22 | 0.07 | 0.26 |
| FA | 0.10 | 0.34 | -0.27 | 0.18 |
| MA | 0.00 | 0.12 | -0.37 | 0.05 |
| GU | -0.04 | 0.14 | -0.48 | |
| **Conduction band alignment (PBE)** | | | | |
| ME | -0.15 | -0.52 | 0.32 | -0.15 |
| FA | -0.03 | -0.09 | 0.19 | 0.33 |
| MA | 0.00 | -0.53 | 0.20 | 0.11 |
| GU | 0.02 | 0.02 | 0.23 | |
| **Conduction band alignment (HSE)** | | | | |
| ME | -0.17 | -0.10 | 0.05 | 0.05 |
| FA | -0.07 | -0.01 | -0.03 | 0.19 |
| MA | 0.02 | -0.14 | 0.02 | 0.04 |
| GU | -0.09 | 0.18 | 0.00 | |

**Table 4. Valence band (VB) level in relation to $MAPbI_3$ and conduction band (CB) relative to $MAPbI_3$ with two different functionals. Trends are mostly consistent and variations in CB alignment is in general smaller than variations in VB alignment.**

*Modelling the maximum efficiency*

The efficiency of the solar cell under operating conditions is modeled by an equivalent circuit depicted in the main paper. The external potential equals the difference in chemical potential for the electrons and holes $eV_{ext} = \mu_e - \mu_h = \Delta\mu$, with $\mu_x$ the chemical potential of electrons and holes respectively. Assuming a constant voltage drop (CVD) model for the heterojunctions, and working on the constant entropy curve, we can derive an expression for the chemical potential difference of electrons and holes. The CVD model implies that no band bending or charge transfer across the interface is taken into account, as this would require explicit information about the material properties of the ETM and HTM beyond the simple level alignment. The assumption of constant entropy means in this case that the only entropy generated is by the excitations, which in turn depends on the carrier masses. Following Ruppel and Wurfel [17] and Osterloh [18], the expression takes the form

$$eV_{ext} = \Delta\mu = E_G - k_B T \ln\left[\frac{2\pi k_B T}{h^2}\frac{4(m_e^* m_h^*)^{3/2}}{n_e n_h}\right] - \Delta U_{htm} - \Delta U_{etm} \quad . \qquad (1)$$

where $V_{ext}$ is the external bias, $\Delta U_{xtm}$is the potential drop in the junction to the electron and hole transporting materials, $m_x^*, n_x$ is the respective carrier mass and density for electrons and holes.

Conservation of current densities assures that $J_{ext} = J_{ph} - J_{rec}$ , that is, the generated current gained from absorption of the photon flux, and loss though carrier recombination. Ideally the only recombinative losses in the system are from black body radiation, although in Silicon impurity assisted (Shockley-Reid-Hall (SRH)) recombination is often dominant. Due to the fact that the perovskites have proven remarkably resilient to material defects, in this work we assume that SRH recombination rates are small in comparison to band-to-band recombination. Previous work by Miyano and co-workers assumed a two diode model where SRH recombination occurs at the interface only.[19] The radiative recombination rate is calculated from

$$RR = e\,\frac{2\pi}{c^2h^3}\int_{E_G}^{\infty}\frac{E^2dE}{\exp\left(E - \Delta\mu/_{k_BT}\right) - 1}$$

Since all the compounds under consideration have a substantial band-gap, we approximate this expression with

$$RR = e\frac{2\pi}{c^2h^3}\exp\left(\Delta\mu/_{k_BT}\right)\int_{E_G}^{\infty}\frac{E^2dE}{\exp\left(E/_{k_BT}\right) - 1} \quad . \qquad (2)$$

Inserting equation (1) in this expression, we have the relation between the radiative recombination rate and the external bias. The expression for the JV characteristics of the PIN junction is

$$J = e\left(N_{ph} - RR(V_{ext})\right) \quad . \tag{3}$$

The number of absorbed photons $N_{ph}$ is calculated assuming all photons above the band-gap are absorbed, that is, the cell is sufficiently thick to eliminate transmission. The solar spectrum used in the numerical simulation is the NREL reference AM1.5 spectrum normalized to 1kW/m$^2$ [ [24] ]. The maximum extractable power density is determined by maximizing $P = V_{ext} \cdot J(V_{ext})$ for the AM1.5 spectrum using parameters from ab-initio calculations, assuming an experimental gap for $MAPbI_3$ of 1.6eV. The full results are presented in table 5. The optimal PCE as a function of band-alignment of HTM and ETM with respect to $MAPbI_3$, for a few representative compounds, is depicted in Fig. 3. Inspiration for the python implementation was acquired from S. J. Byrnes pedagogic IPython notebook[20].

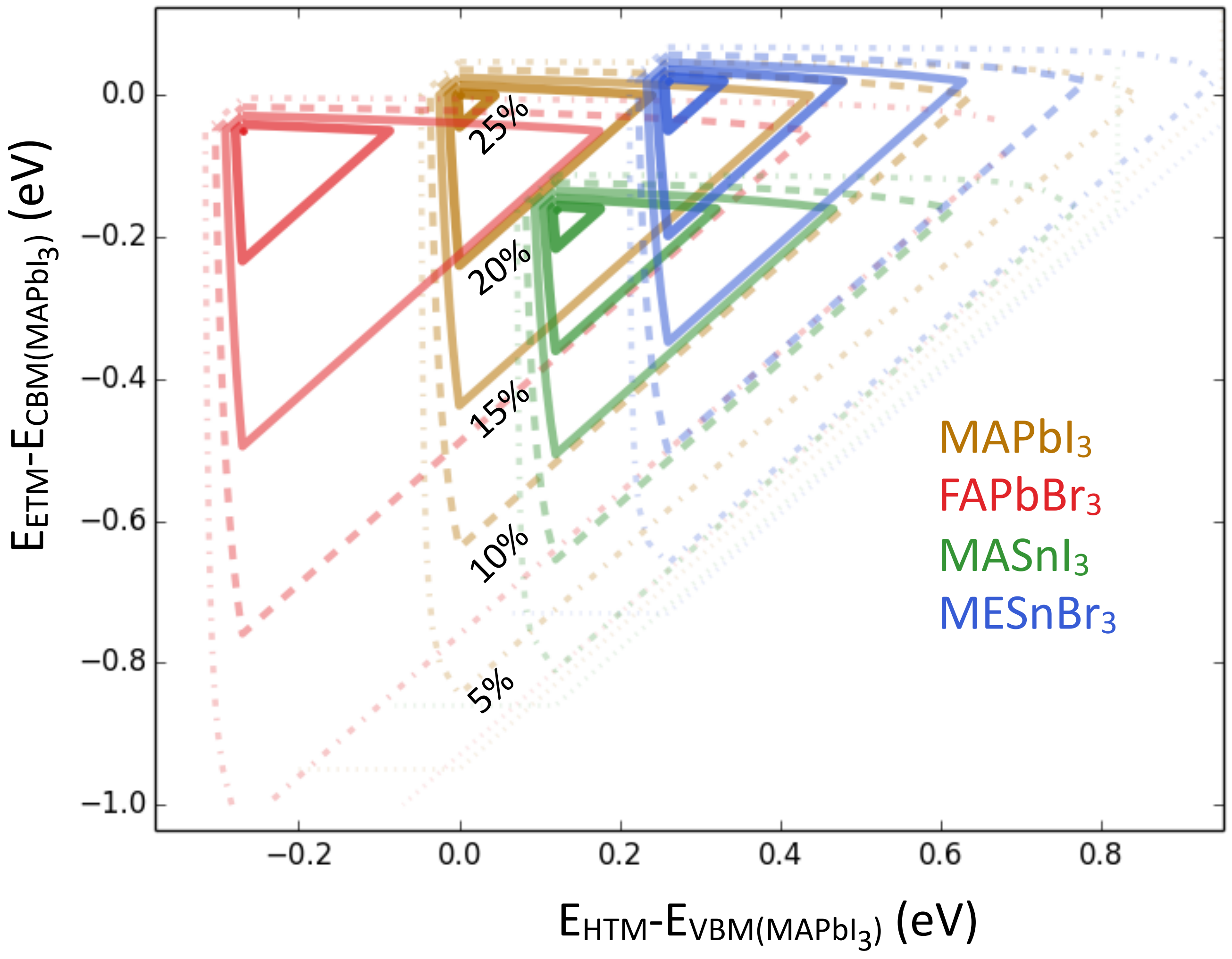


**Figure 3, Efficiency decay as a function of band edge miss-match for a few representative compounds.**

| | Max PCE without entropy contribution (%) | | | | Max PCE including entropy contribution (%) | | | |
|---|---|---|---|---|---|---|---|---|
| | $PbI_3$ | $SnI_3$ | $PbBr_3$ | $SnBr_3$ | $PbI_3$ | $SnI_3$ | $PbBr_3$ | $SnBr_3$ |
| ME | 0.33 | 0.33 | 0.32 | 0.34 | 0.27 | 0.26 | 0.27 | 0.27 |
| FA | 0.33 | 0.33 | 0.27 | 0.31 | 0.27 | 0.27 | 0.24 | 0.26 |
| MA | 0.31 | 0.33 | 0.24 | 0.32 | 0.26 | 0.27 | 0.21 | 0.26 |
| GU | 0.32 | 0.31 | 0.22 | | 0.26 | 0.26 | 0.19 | |

**Table 5. Ideal performance of each cell at 300K without entropy contribution and with entropy contribution. At 300K roughly 15% of the efficiency is lost from the entropy contribution to the chemical potentials of electrons and holes.**

1. Kresse, G. & Hafner, J. Ab initio molecular dynamics for liquid metals. *Phys Rev B* **47,** 558–561 (1993).
2. Kresse, G. & Hafner, J. Ab initio molecular-dynamics simulation of the liquid-metal–amorphous-semiconductor transition in germanium. *Phys Rev B* 14251–14269 (1994).
3. Kresse, G. Efficiency of ab-initio total energy calculations for metals and semiconductors using a plane-wave basis set. *Comp Mat Sci* **6,** 15–50 (1996).
4. Kresse, G. & Joubert, D. From ultrasoft pseudopotentials to the projector augmented-wave method. *Phys Rev B* **59,** 1758–1775 (1999).
5. Perdew, J. P. Generalized gradient approximation made simple. *Phys Rev Lett* **77,** 3865–3868 (1996).
6. Egger, D. A. & Kronik, L. Role of Dispersive Interactions in Determining Structural Properties of Organic-Inorganic Halide Perovskites: Insights from First-Principles Calculations. *Journal of Physical Chemistry Letters* **5,** 2728–2733 (2014).
7. Tkatchenko, A. & Scheffler, M. Accurate Molecular Van Der Waals Interactions from Ground-State Electron Density and Free-Atom Reference Data. **102,** 073005 (2009).
8. Bučko, T., Lebègue, S., Hafner, J. & Ángyán, J. G. Improved Density Dependent Correction for the Description of London Dispersion Forces. *J Chem Theory Comput* **9,** 4293–4299 (2013).
9. Momma, K. & Izumi, F. VESTA: a three-dimensional visualization system for electronic and structural analysis. *J Appl Crystallogr* **41,** 653–658 (2008).
10. Björkman, T. CIF2Cell: Generating geometries for electronic structure programs. *Computer Physics Communications* **182,** 1183 (2011).
11. Galkowski, K., Mitioglu, A. & Miyata, A. Determination of the exciton binding energy and effective masses for methylammonium and formamidinium lead tri-halide perovskite semiconductors. *Energy Environ. Sci.* **9,** 962–970 (2016).
12. Menéndez-Proupin, E., Palacios, P., Wahnón, P. & Conesa, J. C. Self-consistent relativistic band structure of the CH3NH3PbI3perovskite. *Phys Rev B* **90,** 045207 (2014).
13. Feng, J. & Xiao, B. Effective Masses and Electronic and Optical Properties of Nontoxic MASnX 3(X = Cl, Br, and I) Perovskite Structures as Solar Cell Absorber: A Theoretical Study Using HSE06. *J Phys Chem C* **118,** 19655–19660 (2014).
14. Umari, P., Mosconi, E. & De Angelis, F. Relativistic GW calculations on CH3NH3PbI3 and CH3NH3SnI3 Perovskites for Solar Cell Applications. *Sci. Rep.* **4,** 4467 (2014).
15. Heyd, J., Scuseria, G. E. & Ernzerhof, M. Erratum:'Hybrid functionals based on a screened Coulomb potential'[J. Chem. Phys. 118, 8207 (2003)]. *J. Chem. Phys.* **124,** 219906 (2006).
16. Ahmed, T. *et al.* Optical Properties of Organometallic Perovskite: An ab initio Study using Relativistic GW Correction and Bethe-Salpeter Equation. (2014).
17. Ruppel, W. & Wurfel, P. Upper limit for the conversion of solar energy. *Electron Devices* (1980).
18. Osterloh, F. E. Maximum theoretical efficiency limit of photovoltaic devices: effect of band structure on excited state entropy. *J. Phys. Chem. Lett.* **5,** 3354–3359 (2014).

19. Miyano, K., Tripathi, N. & Yanagida, M. Lead Halide Perovskite Photovoltaic as a Model p–i–n Diode. *Acc Chem Res* **49,** 303–310 (2016).
